# Real time complete Stokes polarimetric imager based on a linear polarizer array camera for tissue polarimetric imaging

**DOI:** 10.1364/BOE.8.004933

**Published:** 2017-10-10

**Authors:** Ji Qi, Chao He, Daniel S. Elson

**Affiliations:** 1 Hamlyn Centre for Robotic Surgery, Institute of Global Health Innovation, Imperial College London, Exhibition Road, London SW7 2AZ, UK; 2 Department of Surgery and Cancer, Imperial College London, Exhibition Road, London SW7 2AZ, UK

**Keywords:** (120.5410) Polarimetry, (170.6935) Tissue characterization

## Abstract

Tissue polarimetric imaging measures Mueller matrices of tissues or Stokes vectors of the emergent light from tissues (normally using incidence with a fixed polarization state) over a field of view, and has demonstrated utility in a number of surgical and diagnostic applications. Here we introduce a compact complete Stokes polarimetric imager that can work for multiple wavelength bands with a frame-rate suitable for real-time applications. The imager was validated with standard polarizing components, and then employed as a polarization state analyzer of a Mueller imaging polarimeter and a standalone Stokes imaging polarimeter respectively to image the process of dehydration of bovine tendon tissue. The results obtained in this work suggested that the polarization properties of the samples rich of collagen fibres can change with the degree of dehydration, and therefore, dehydration of the samples prepared for polarimetric imaging (e.g. polarimetric microscopy) should be carefully controlled.

## 1. Introduction

Polarization imaging has aroused interest in the field of tissue imaging since it can be a powerful tool to reveal morphological, structural and compositional information of tissue. Tissue polarimetric imaging measures (complete/partial) Mueller matrices of biological tissues or (complete/partial) Stokes vector of the emergent light from tissues (normally using incidence with a fixed polarization state) over a field of view. Tissue Mueller polarimetric imaging has demonstrated utility in a number of surgical and diagnostic applications [1], like cancer diagnosis [2, 3], non-staining pathology inspection [4–6], partial bladder obstruction diagnosis [7] etc. Stokes polarimetric imaging was also used together with linearly polarized or circularly polarized illumination to reveal subsurface tissue structural information that could be useful for diagnosis [8, 9], and cancerous tissue identification [10]. Many polarimetric imaging and devices are emerging to pave the way for the translation of polarimetric imaging techniques to surgery and tissue diagnosis [11–14].

A Stokes polarimetric imager is a core device for Stokes polarimetric imaging techniques, and is also an essential component of a Mueller imaging polarimeter known as the polarization state analyzer (PSA for short). A real time, lightweight and compact Stokes polarimetric imager that can work under multiple wavelength-bands in the visible range is desirable since it would be easier to integrate such an imager with endoscopes and microscopes to do *in vivo* polarimetric imaging. As a fast PSA, it can also accelerate the acquisition speed and pave the way for fast Mueller polarimetric imaging.

Stokes polarimetric imagers can be constructed with mechanically rotating waveplates or linear polarizers. A polarimeter with mechanically moving parts has disadvantages like relatively slow speed, vibration, shorter life etc., so mechanically moving parts are avoided where possible when polarimetric imagers are designed. Stokes imagers with two temperature controlled liquid crystal viable retarders (LCVRs) working together to time-sequentially generate four pairs of phase retardance to reconstruct one Stokes image have been demonstrated [15]. At least four images need to be acquired, and it also usually takes the LCVRs tens of milliseconds to switch their retardance once, which limits the acquisition speed of this kind of device. In addition, a temperature controlled LCVR has a relatively small aperture size and long length (a typical aperture size and length are 10mm and 30mm respectively), and therefore two LCVRs used in series in an imaging system may restrict the amount of light (especially for off-optical-axis object points) which passes through the imaging system, resulting in artefacts like strong vignetting, reduction of field of view, restricted transmittance etc. Using dual photo-elastic modulators (PEMs) instead of LCVRs may make the acquisition much faster [16, 17]. Like LCVRs, PEMs can also generate variable retardance. PEMs have a very high modulation frequency determined by the piezoelectric material in the transducer. The Stokes polarimeter based on PEMs entails a high-speed camera and the image has to be obtained with low exposure time on the scale of several micro-seconds, in addition to careful synchronization of the camera and PEMs. It would be difficult to use this approach for applications requiring several to tens of milli-seconds exposure time which is typical in endoscope imaging.

The integration of a pixelated micro-linear-polarizer array (LPA) directly onto an image sensor has become achievable nowadays to measure the polarization states in a single snapshot. However, the vast majority of this kind of devices are only sensitive to linear polarization. There are only several exceptions which were based on customized pixelated micro-elliptical-polarizer arrays with liquid-crystals and wire grid linear polarizers [18–20]. The retardance of these micro-elliptical polarizer arrays, the same as normal elliptical or circular polarizers, are designed and optimized for only a single narrow wavelength-band, e.g. only working at 580nm in [20], 760nm in [18] and 500nm in [19] (It should be noted that an ill-optimized polarimeter is not calibratable due to the magnified random error that presents in calibration measurements).

Here we demonstrate a compact complete Stokes polarimetric imager based on a LPA camera and a LCVR. The imager can work for multiple wavelength bands with the frame rate suitable for real time applications. It has been validated with standard polarizing components, and was then employed as the PSA of a Mueller imaging polarimeter and a standalone Stokes imaging polarimeter respectively to image the process of dehydration and associated optical clearing of bovine tendon tissue.

## 2. System design and construction

### 2.1 Stokes vectors and Mueller matrices

A Stokes vector ***S*** is a four-element vector used to characterize polarization state of light. According to its definition, ***S*** can be determined by a set of radiometric intensity measurements using “horizontal” (*I_H_*), “vertical” (*I_V_*), + 45° (*I*_45_), −45° (*I*_-45_) linear, as well as left (*I_L_*) and right (*I_R_*) circular polarization analysers.

$$S=\left[\begin{array}{c} S_{0}\\ S_{1}\\ S_{2}\\ S_{3} \end{array}\right]=\left[\begin{array}{c} I_{H}+I_{V}\\ I_{H}-I_{V}\\ I_{\text{45}}-I_{\text{-45}}\\ I_{L}-I_{R} \end{array}\right]$$ (1)

The degree of polarization (*DOP*) of light refers to the intensity of the polarized portion of the light out of its total intensity, and can be calculated using the following equation,

$$DOP=\frac{\sqrt{{S_{1}}^{2}+{S_{2}}^{2}+{S_{3}}^{2}}}{S_{0}}$$ (2)

The *DOP* is 1 for fully polarized light, 0 for unpolarized light, and in the range 0-1 for partially polarized light.

The polarization state of light may be transformed during polarized light-sample interactions, depending on the polarization properties of that sample. The mathematical description of the transformation is a Mueller matrix defined in the Stokes vector space. A Mueller matrix *M* can transform an input Stokes vector ***S****_in_* to an output Stokes vector ***S****_out_*, expressed as

$$S_{out}={MS}_{in}=\left[\begin{array}{cccc} m_{11} & m_{12} & m_{13} & m_{14}\\ m_{21} & m_{22} & m_{23} & m_{24}\\ m_{31} & m_{32} & m_{33} & m_{34}\\ m_{41} & m_{42} & m_{43} & m_{44} \end{array}\right]S_{in}$$ (3)

A Mueller matrix is a complete characterization of the polarization properties of a sample.

### 2.2 Experimental setup

**Fig. 1:**
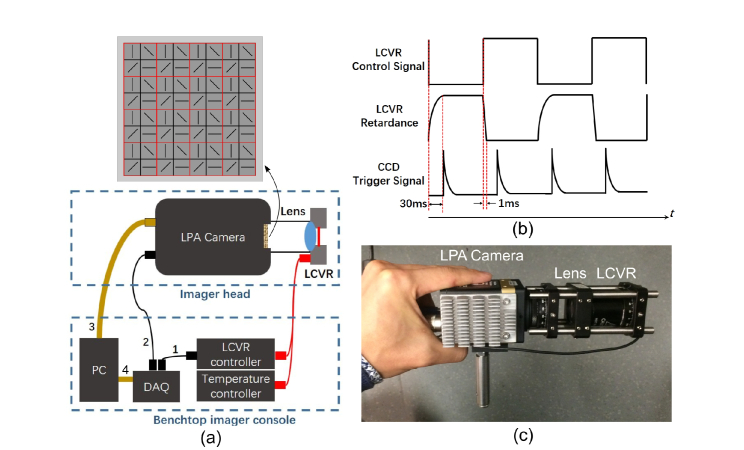
(a) Experimental set-up of the Stokes polarimetric imager including a handhold-able imager head and a benchtop imager console. The imager head consisted of a LPA camera with pixelated linear polarizer array on its focal plane, an objective lens and a temperature controlled LCVR. In the console, a DAQ card generated two synchronized signals - a LCVR control signal sent to the LCVR controller via Cable ‘1’ and a CCD trigger signal sent to the LPA camera via Cable ‘2’; A PC communicated with the LPA camera via Cable ‘3′ and the DAQ via Cable ‘4’. (b) Timing diagram of LCVR control signals, the LCVR retardance and CCD trigger signals. The time delay between the CCD trigger pulses and the LVCR control signal was set as 30 ms; (c) Photo of the Stokes polarimetric imager head.

As displayed in Fig. 1(a)

, the full Stokes polarimetric imager consisted of a camera with pixelated linear polarizer array on its focal plane (PolarCam, 4D Technology Inc., named “LPA camera” in this paper), an objective lens (6 mm focal length), a temperature controlled liquid crystal variable retarder (LCC1111T-A, Thorlabs Inc, LCVR for short), and a Data Acquisition Card (USB-6211, National Instruments, DAQ for short). The LPA camera was originally modified from an industrial CCD camera with 1384*1208 pixels by its manufacturer. Each CCD pixel was aligned with the individual linear polarizer element of the LPA array. Every four adjacent CCD pixels enclosed by the red square in Fig. 1 constitute a super-pixel where the CCD pixels comprising the super-pixel were covered by 0°, 45°, 90°, and 135° wire-grid linear polarizers. The size of an individual pixel is 7.4 um × 7.4 um. A super-pixel thus corresponds to a 14.8 um × 14.8 um area. The spatial resolution of this Stokes imager is determined by that of the LPA camera, which was discussed in details in [21]. The maximum frame rate of this LPA camera can reach 35 frames/s. This camera can resolve the first three elements of a Stokes vector and its nominal Data Reduction Matrix (DRM for short, also called Polarization State Analyzer (PSA) instrumental matrix in Mueller polarimetry) is:

$${DRM}_{cam}=\left[\begin{array}{cccc} 1 & 1 & 0 & 0\\ 1 & 0 & 1 & 0\\ 1 & -1 & 0 & 0\\ 1 & 0 & -1 & 0 \end{array}\right]$$ (4)

A nematic liquid crystal based variable retarder was employed in this work in order to determine a complete Stokes vector. LCVRs have advantages including fast switching time (typically ranging from several to tens of millisecond), no moving parts, no high driving voltages, and suitability for imaging with arbitrary exposure time because the retardance of LCVRs is stable during exposure. Since LCVRs are sensitive to the environmental temperature, temperature control is required for polarimetric measurement. A temperature control unit (TC200, Thorlabs Inc.) was used in this work to prevent interference from variations in room temperature and set at 35°C. The Mueller matrix of the LCVR is represented by:

$$\begin{array}{c} M_{LCVR}(\theta ,\delta )=Rot(2\theta )M_{WP0}(\delta )Rot(-2\theta )\\ M_{WP0}(\delta )=\left[\begin{array}{cccc} 1 & 0 & 0 & 0\\ 0 & 0 & 0 & 0\\ 0 & 0 & \cos\delta & -\sin\delta \\ 0 & 0 & \sin\delta & \cos\delta \end{array}\right]\;Rot(X)=\left[\begin{array}{cccc} 1 & 0 & 0 & 0\\ 0 & \cos X & -\sin X & 0\\ 0 & \sin X & \cos X & 0\\ 0 & 0 & 0 & 1 \end{array}\right] \end{array}$$ (5)

where *δ* and *θ* are the magnitude of its linear retardance and fast axis orientation respectively. The LCVR was modulated between two retardance states in the temporal domain, and one image was acquired by the LPA camera per retardance state, so that the *DRM* of the Stokes polarimetric imager in Eq. (6) is invertible.

$$DRM=\left[\begin{array}{c} {DRM}_{cam}M_{LCVR}(\theta ,\delta_{1})\\ {DRM}_{cam}M_{LCVR}(\theta ,\delta_{2}) \end{array}\right]$$ (6)

The complete Stokes vector ***S*** can thus be obtained according to the following equation,

$$\begin{array}{l} S=DRM^{-1}I_{n}\\ I_{n}={\left[I_{\left(0,\delta 1\right)},I_{\left(45,\delta 1\right)},I_{\left(90,\delta 1\right)},I_{\left(-45,\delta 1\right)},I_{\left(0,\delta 2\right)},I_{\left(45,\delta 2\right)},I_{\left(90,\delta 2\right)},I_{\left(-45,\delta 2\right)}\right]}^{T} \end{array}$$ (7)

The superscripts ^−1^ and ^–^*^T^* denote Moore–Penrose pseudoinverse and matrix transpose respectively, and ***I****_n_* is a column vector containing the intensity readings from each pixel of a super-pixel under the two retardance states *δ*_1_ and *δ*_2_ respectively.

LCVRs generally produce high retardance with low driving voltage and low retardance with high driving voltage. The switching time from a high to a low retardance state is less than 1 ms, but it typically takes much longer from a low to a high retardance state, as shown in the diagram in Fig. 1(b). It is noted that higher working temperatures result in shorter switching times. The retardance modulation of the LCVR and the image acquisition by the LPA camera were externally synchronized using two electric trigger pulses generated by the DAQ card. A time delay was set between the two pulses to make sure the LPA camera started acquisition after the retardance of LCVR was fully settled, as shown in Fig. 1(b). The determination of the delay is specified in Section 2.4.

The photo of this Stokes polarimetric imager was shown in Fig. 1(c). The imager is compact and handholdable. The angle-of-view of this imaging system was 12° which was mainly determined by the objective lens used. It can be increased by using an objective with shorter focal length.

### 2.3 Polarimetric system optimization

**Fig. 2:**
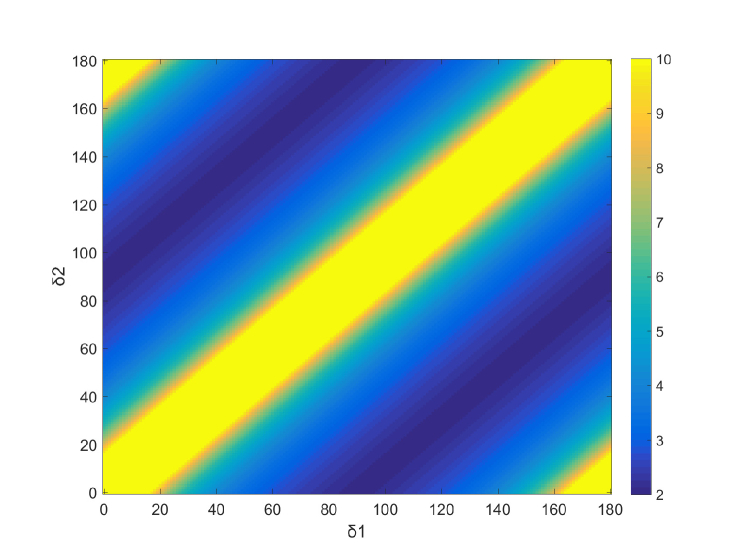
The condition number map of the *DRM* of the Stokes polarimetric system. The horizontal axis represents *δ*_1_ and the vertical axis refers to *δ*_2_ of the LCVR. The map does not change with *θ*. The minimum condition number is obtained when the difference of *δ*_1_ and *δ*_2_ reaches 90°.

It is necessary to optimize the *DRM* of the Stokes polarimetric imager in order to minimize the error propagating from the measured radiometric vector ***I****_n_* to the reconstructed Stokes vector ***S*** in Eq. (7). According to Eq. (6) *DRM* is a function of *θ*, *δ_1_* and *δ_2_*. The optimization can be conducted by finding the optimal *θ*, *δ_1_* and *δ*_2_ so that the 2-norm condition number of the *DRM* reaches its minimum [22, 23]. It is found that the azimuthal orientation of the LCVR *θ* does not have any effect on the condition number. The condition number of the *DRM* is a function of the difference of *δ*_1_ and *δ*_2_ of the LCVR. The condition number map where the horizontal and vertical axes refer to *δ*_1_ and *δ*_2_ respectively is plotted in Fig. 2

(this map does not change with the value of *θ*). The minimum condition number 2 is obtained when the difference reaches 90°, that is,

$$\left|\delta_{1}-\delta_{2}\right|=90{}^{\circ}$$ (8)

### 2.4 Characterization of the LCVR

**Fig. 3:**
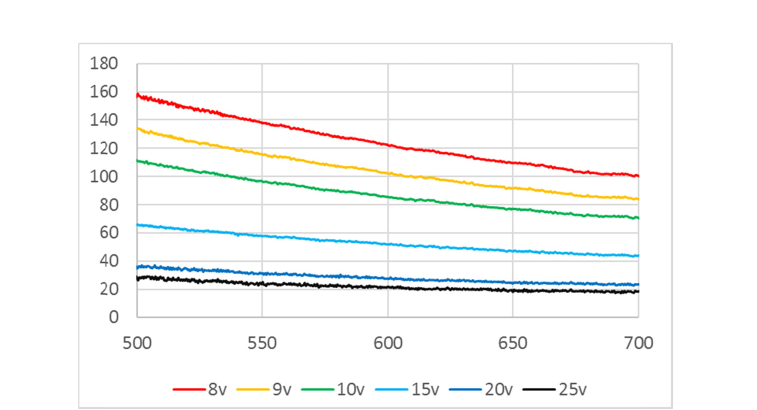
The retardance spectra of the LCVR with the drive voltages set to 8 V, 9 V, 10 V, 15 V, 20 V, 25 V. The horizontal axis and vertical axis represent the wavelength in nanometer and the retardance in degree respectively.

Since the retardance of the LCVR at a certain drive voltage is wavelength dependent, the spectral properties of the LCVR were characterized to achieve the two optimal retardance states of the LCVR at the 35°C working temperature. The characterization set-up consisted of a broadband halogen light source (HL-2000, Ocean Optics), a horizontal linear polarizer, the LCVR with its fast axis orientated at + 45°, a rotatable linear analyzer and a spectrometer (HR-4000CG-UV-NIR, Ocean Optics). Two spectra with the linear analyzer horizontal (*HH*(*λ*)) and vertical (*HV*(*λ*)) were recorded for each LCVR drive voltage (8 V, 9 V, 10 V, 15 V, 20 V and 25 V). The retardance spectra *δ*(*λ*) were then calculated according to the equation *δ*(*λ*) = cos^−1^[*HH*(*λ*)-*HV*(*λ*))/(*HH*(*λ*) + *HV*(*λ*)] given in [24] and displayed in Fig. 3

. In general, under a drive voltage, the LCVR exhibited lower retardance at long wavelengths, and comparably higher retardance at short wavelengths. The LCVR also demonstrated stronger wavelength dispersion when working at lower voltages.

Two wavelength-bands (546 nm central wavelength with about 20 nm bandwidth, and 628 nm with about 20 nm bandwidth) generated by a lamp based light source (Lumen-Pro200, Prior Scientific) incorporating two band pass filters were involved in this work in order to demonstrate that this Stokes imager could work at multiple wavelength-bands. At 546 nm, the highest drive voltage (25 V) had the lowest retardance 24°. According to Eq. (8), 114° retardance of the LCVR should be used to satisfy the optimal setting as discussed in the last section. Referring to Fig. 3, the corresponding drive voltage was near 9 V. Interpolation was conducted to obtain the specific voltage corresponding to 114°, which was found to be 9.17 V. The switching time of the LCVR was also characterized using the light source (546/20 nm band), a horizontal linear polarizer, the LCVR with arbitrary fast axis orientation (but not parallel or orthogonal to the first linear polarizer), a vertical linear analyzer, a focusing lens, a photodiode (DET36A/M, Thorlabs, rise time 14 ns) and the DAQ card (NI-USB6211, sampling rate up to 250 kHz) to read and record the analogue signal from the photodiode. It took the LCVR 25 ms to switch from 24° to 114°, and 0.7 ms from 114° to 24°. The same procedure was repeated for the 628/20 nm band, and the voltage pair was selected as 8.26 V and 25 V corresponding to 110° and 20° retardance of the LCVR respectively. The switch time was measured as 20 ms (from 20° to 110°) and 0.5 ms (110° to 20°). Therefore, the time delay between the trigger pulses for the LPA camera and LVCR was set as 30 ms for all the experiments (to make sure that each Stokes image corresponded to the same sampling time).

### 2.5 Calibration

For calibration, this Stokes imager was then adapted to a Mueller polarimeter as the PSA. The polarization state generator (PSG) of the Mueller polarimeter consisted of a fixed 0° linear polarizer and a rotating quarter waveplate rotated to −45°, 0°, 30°, 60° (optimized in [22]) using a motorized rotation stage (PRM-Z1, Thorlabs. Inc.). The entire Mueller polarimeter was then calibrated pixel-wisely according to Eigenvalue calibration method [25] by using a horizontal linear polarizer, a vertical linear polarizer and a quarter waveplate orientated at about 30° as suggested in [23], and the actual instrumental matrices of the PSA and the PSG could be obtained. The PSA instrumental matrix was the *DRM* of the Stokes imager. An advantage of using this calibration method is that it does not require a pre-calibrated PSG. The condition number of the calibration determined *DRM* is 2.2, very close to 2, the optimal value in theory. The minor increase should mainly arise from the relatively low extinction ratio of the micro-linear polarizers in the LPA as well as cross talk among pixels due to internal scattering between the LPA array and the image sensor.

## 3. Results

### 3.1 Validation experiment

**Fig. 4:**
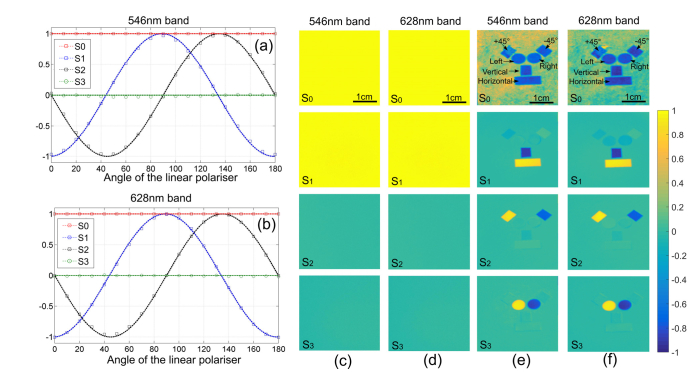
The variation in the four elements of Stokes vectors of a linear polarizer rotated from 0° to 180° with a step of 10°(a) 546 nm band and (b) 628 nm band. The squares and circles in (a) and (b) represent the data acquired using the Stokes polarimetric imager. The dash lines in (a) and (b) stand for the theoretically prediction of the Stokes vectors; (c) the Stokes polarimetric image of a linear polarizer at 546 nm band and (d) 628 nm band; (e,f) the Stokes polarized image of the sample with horizontal, vertical, + 45°, −45° linear polarizers and left and right circular polarizers on the top of a piece of paper under two narrow band light sources. A Stokes polarimetric video was also generated when this sample was moving, and was shown in Visualization 1.

The Stokes polarimetric imager was validated using a linear polarizer rotated from 0° to 180° in 10° steps for a 546 nm and 628 nm narrowband light source. The Stokes polarimetric images were reconstructed according to Eq. (7), and normalized via pixel-wise division by the first element of the reconstructed Stokes vector. By processing the 30 × 30 super-pixel region (which by careful visual inspection contained no bad pixels) in the centre of the field of view, the variation in the four elements of Stokes vectors with the linear polarizer orientation was calculated and plotted (as represented by squares and circles) in Fig. 4

for the (a) 546 nm band and (b) 628 nm band. Theoretically, the *S*_1_ and *S*_2_ curves should be two sinusoidal functions with 90° phase difference, and *S*_3_ should always be zero, as shown by the dash lines in Fig. 4(a) and 4(b). The measured Stokes vectors were close to the theoretical expectation. The maximum elemental error of the reconstructed Stokes vectors was smaller than 0.02 for the 546 nm band and the 628 nm band without considering bad pixels and pixels affected by dust. The Stokes polarimetric images (consisting of 690 × 600 super-pixels in each element image, all the Stokes element images were divided by the *S*_0_ image pixelwisely) of a horizontal linear polarizer at 546 nm and 628 nm were displayed in Fig. 4(c) and 4(d) respectively. The Stokes vector of a perfect horizontal linear polarizer is [1, 1, 0, 0]. The mean Stokes vector of the entire image was calculated as [1, 1.005, 1.003, 0.988] for the 546 nm band with the standard deviation 0, 0.012, 0.0144, 0.018 for *S*_0,1,2,3_ respectively, and [1, 0.995, 0.987, 0.987] for 628 nm band with the standard deviation 0, 0.015, 0.005, 0.019 for *S*_0,1,2,3_.

As demonstrated in Fig. 4(e), 4(f), a sample was also used to validate the imaging function, and consisted of six standard polarizing components including an (approximately) horizontal (lower rectangle), vertical (middle rectangle), + 45° (rectangle at the upper right), −45° linear polarizing film (upper left rectangle) and left (right-hand circle) and a right (left-hand circle) circular polarizers on the top of a depolarizing piece of paper . The sample was illuminated from the side without the polarizer (that is, the illumination went through the paper), and the Stokes imager acquired images from the side with the polarizer for the two wavelength bands. The light transmitted by different parts of the sample thus has known polarization states.

Reconstruction of Stokes polarimetric images was based on Eq. (7). A Stokes polarimetric image with 690 × 600 super-pixels in each element image took 0.79 seconds using MATLAB 2011 in a Windows laptop with Intel Core i5-3320 and 16GB RAM. Reconstruction of Stokes polarimetric images only involves pixelwise multiplication of a 4 × 8 *DRM*^−1^ matrix and a 8 × 1 ***I****_n_* matrix according to Eq. (7), and can be accelerated to be real time by using more efficient programming languages or/and parallel computing techniques. The images of the second, third and the fourth elements of the Stokes polarimetric image were then normalized by dividing the image of the first element. The image of the first element, which was unnormalized, represented polarization insensitive transmittance intensity, and since the polarizing components have absorption they could be differentiated from the paper background in the first element images in Fig. 4(e) and 4(f). In the image of the second element, the horizontal and vertical polarizers had opposite values as expected, and were easily identified from the background, as the other polarizers and depolarizing paper demonstrated values close to zero. For similar reasons, the + 45° and −45° linear polarizers were highly visible on the images of the third element, and the left and right circular polarizers demonstrated sharp difference from the others on the image of the fourth element. The same sample was also imaged with 628 nm light, and it has been demonstrated that this Stokes imager can work well for both wavelengths. It is noted that the circular polarizers used here consisted of a quarter waveplate and a linear polarizer. The retardance of the quarter waveplate in the circular polarizers deviated from 90° at 628 nm. As a result, the circular polarizers became effective elliptical polarizers and appeared at the *S*_1_ image in Fig. 4(f). It is also noted that the “+45°” and “-45°” linear polarizers were approximately aligned as + 45° and −45°, when we prepared this sample. Therefore, minor cross-talk between *S*_1_ and *S*_2_ were expected.

A Stokes polarimetric video was also generated when this sample was moving, and was shown in Visualization 1. The video frame rate reached 16 frames/second with 15 ms exposure time for each individual image. The frame rate can be further increased by reducing exposure time or enabling the multiple-tap function of the camera (an image sensor is divided into several regions, so that the pixel values in each region can be readout in parallel). It has been illustrated in this experiment that the Stokes polarimetric imager can be used to determine the Stokes vector images in real-time under multiple wavelength-bands. This imager can easily work with other wavelength-bands by simply changing the drive voltages for optimization purposes.

### 3.2 Using the imager as the PSA of a Mueller polarimeter to image the dehydration process of tendon tissue

In this experiment, the Stokes polarimetric imager was employed as the PSA of a transmission Mueller polarimeter to accelerate acquisition time for Mueller polarimetric imaging. The setup was the same as the Mueller polarimeter described in Section 2.4 and used the 546/20 nm band light as the source. The acquisition time for a Mueller polarimetric image was about 9 seconds. Over 95% of the acquisition time was spent waiting for the acceleration, rotation and deceleration of the slow motorized rotation stage utilized here (PRM-Z1, Thorlabs Inc.). This imaging Mueller polarimeter can be further accelerated if a faster rotation stage (like DDR100/M, Thorlabs) or a different configuration of PSG (e.g. variable retarder based) were used. If LCVRs were also used in the PSG, the acquisition time of a Mueller polarimetric image can be within 0.32 second (10 ms exposure time for raw images and 30 ms LCVR response time, and eight raw images and eight LCVR switches are needed). This Mueller imaging polarimeter based on the real-time Stokes imaging polarimeter was sufficient for us to monitor the dehydration process of a slice of bovine tendon (thickness about 0.5 mm, cut from *ex vivo* bovine tendon) at a reasonable frame-rate. The sample was exposed in the air and underwent evaporation, resulting in dehydration over a period of about 16 minutes. Mueller polarimetric images were acquired every minute to monitor the change of polarization properties of the sample, and a video file that contains all of the acquired Mueller images is shown in Visualization 2. The Mueller polarimetric images were then decomposed based on Lu-Chipman’s polar decomposition method, and the magnitude of diattenuation, total depolarization, the magnitude of retardance and fast axis orientation images were then reconstructed [26, 27].The reconstruction and decomposition of a Mueller polarimetric image took 7.96 seconds and 112.86 seconds respectively using MATLAB 2011 in the laptop with Intel Core i5-3320 and 16GB RAM.

**Fig. 5:**
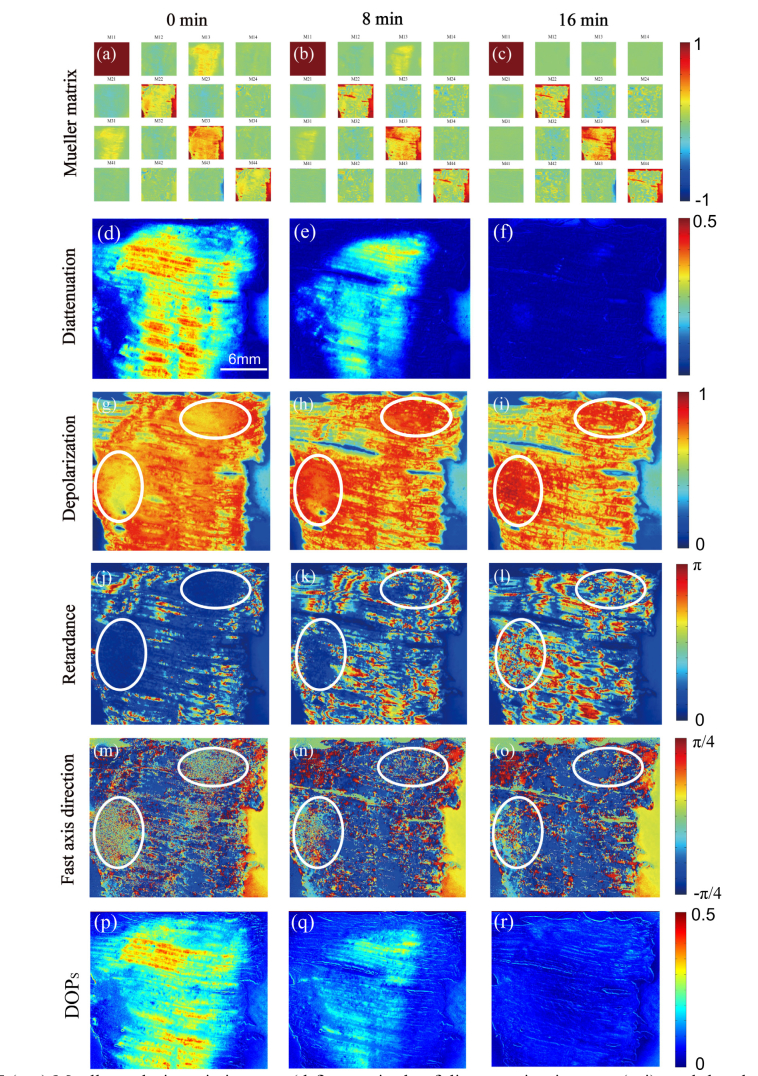
(a-c) Mueller polarimetric images, (d-f) magnitude of diattenuation images, (g-i) total depolarization images, (j-l) magnitude of retardance images, (m-o) fast axis orientation images, (p-r) the degree of polarization images reconstructed from Stokes images with unpolarized illumination denoted by *DOP_S_* of the bovine tendon sample. The images in the first, second and third column were obtained at 0min, 8min and 16min respectively. (d-r) and all the element images in (a-c) share the same scale bar specified in (d). Visualization 2, Visualization 3, Visualization 4, and Visualization 5 recorded the change of Mueller polarimetric images, and the diattenuation, retardance and depolarization images in the 16 min.

The results acquired at 0, 8 and 16 minutes are displayed in Fig. 5

. The Mueller matrix of the tendon was essentially symmetrical and remained symmetrical during the dehydration process, as demonstrated in Fig. 5(a)-5(c). More details can be found from the decomposed images. The diattenuation magnitude of the sample was strong at 0 minutes, as observed in Fig. 5(d), which is expected to arise mainly from scattering by well-aligned collagenous fibrillar structure of the tendon [28]. In the dehydration process, diattenuation dramatically decreased as shown in Fig. 5(d)-5(f). Tissue dehydration is known to be an important mechanism of tissue optical clearing, as this process normally provides a refractive-index matching effect due to reduction of water content in the interstitial space, which reduces the overall thickness of tissue and increases the structural density, resulted in weaker scattering from tissue [29]. The reduction of scattering-induced tissue diattenuation during dehydration can thus be anticipated.

As with dehydration, retardance generally became more prominent in Fig. 5(j)-5(l), and became the dominant polarization property when the sample was highly dehydrated at 16 min, as also found in the fast axis orientation images demonstrated in Fig. 5(m-o). The two areas indicated by white ovals in Fig. 5(m)-5(o) were noisy at 0 min due to the very low tissue retardance (the equation to derive fast axis orientation is close to singular) as can be seen in Fig. 5(j). The noise gradually disappeared with dehydration as shown in Fig. 5(n), and finally demonstrated a uniform distribution in accordance with the fast axis orientation of the surrounding tissues at 16 min. The increase in retardance in the dehydration process should be closely related to the increase of fibrous structure density of the tendon which enhanced tissue birefringence.

The depolarization images are demonstrated in Fig. 5(g)-5(i). Since the thickness of the tendon slice was more uniform at 0 min, the depolarization of the sample was relatively homogeneous. As with dehydration, the depolarization image of the sample appeared to be increasingly textured and heterogeneous, resulting from the shape and local structure density of the sample changes. Depolarization in some regions of the sample decreased due to weaker scattering. However, it is noted that depolarization does not only depend on scattering (by both the well-aligned fibrillar structures and other tissue structures and scatterers) but also on tissue retardance. Depolarization rises with increasing tissue retardance, as previously reported in [2, 30, 31]. Although scattering of the sample became weaker with dehydration, the depolarization still increased in some parts of the sample with increasing retardance, represented by the two areas indicated by the white ovals. In these areas, retardance was more a dominant mechanism to affect depolarization than scattering.

It is noted that the change of polarization properties of the sample was continuous and smooth, which can be observed from the Visualization 2, Visualization 3, Visualization 4, and Visualization 5 that recorded the change of Mueller polarimetric images, and the diattenuation, retardance and depolarization images in the 16 min.

Since histological staining techniques require time and elaborate sample preparation by a well-trained and experienced pathologist for diagnosis, polarimetric microscopy can potentially be employed to image unstained thin tissue cuts for rapid and low cost pathological inspection [5, 6, 32–37], especially for diagnosis related to tissue fibrosis [38, 39]. In this work, it is found that the polarization properties of the samples rich of collagen fibres changed with the degree of dehydration. Therefore, the dehydration of the samples prepared for polarimetric microscopy should be carefully controlled to guarantee consistency among inter- and intra- sample inspection.

Although tissue optical clearing has demonstrated its potential biomedical applications, investigation of its mechanism is still underway [29, 40]. Advanced sensing or imaging techniques are desirable to reveal the microstructural information of the tissues during the optical clearing process [41]. Transmission electron microscopy (TEM) is a common technique to examine the change of tissue microstructures in optical clearing [41]. The results obtained here demonstrated that tissue polarization properties especially tissue diattenuation were sensitive to dehydration induced microstructure changes for tissues rich of fibrous structures, and suggested that polarimetric imagers have potential to become an alternative tool of much lower cost and of higher acquisition speed to characterize the tissue microstructures. Here we explored whether it is possible to use the Stokes polarimeter alone - rather than as part of a Mueller polarimeter - to assess tissue diattenutation, since Stokes polarimetric imaging simpler, particularly in terms of acquisition time, device volume and weight etc., and is thus easier to adapt to e.g. polarimetric endoscopes and microscopes.

The three fundamental polarization properties are diattenuation, retardance and depolarization [1] and a Mueller matrix can always be decomposed into diattenuation, retardance and depolarization matrices. According to the definition of these three fundamental polarization properties, diattenuation is the property that can increase the DOP of light during light-sample interaction, and the extent of the increase in DOP during the interaction depends on the magnitude of diattenuation. Therefore, using unpolarized illumination (*DOP* = 0), the magnitude of diattenuation can be assessed by calculating the DOP of the emergent light from a sample. As an initial exploration of this, the Stokes measurements of this tendon slice were calculated based on the Mueller matrix images acquired multiplying the incident Stokes vector [1, 0, 0, 0], and the DOP images of the emergent Stokes measurements denoted by *DOPs* were displayed in Fig. 5(p)-5(r). It can be found that *DOP_S_* images demonstrate a similarity to the diattenuation image, and the change of *DOP_S_* with dehydration was highly consistent with diattenuation.

### 3.3 Using the imager alone to image the dehydration process of tendon tissue

**Fig. 6:**
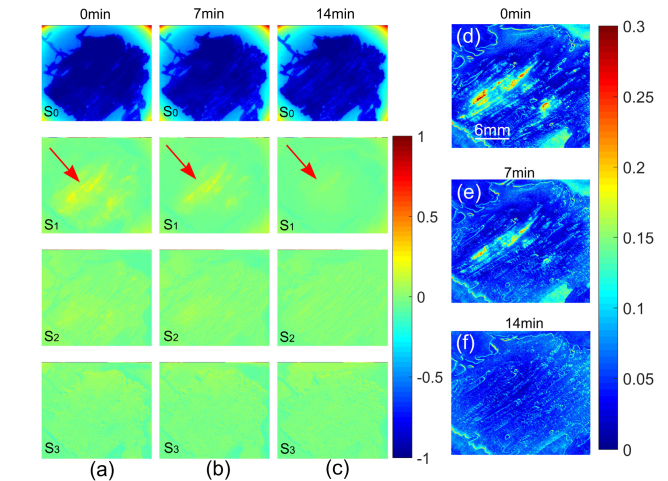
(a-c) Stokes polarimetric images of another tendon sample acquired at 0min, 7min and 14min, (d-f) *DOP_S_* images of the sample at 0min, 7min and 14min respectively. (d-f) and all the element images in (a-c) share the same scale bar specified in (d).

Another slice of bovine tendon was prepared (thickness about 0.7 mm, cut from *ex vivo* bovine tendon). In this experiment, unpolarized light (the 546 nm band) was used to illuminate the sample, and the Stokes polarimetric imager was used to image the sample in a transmission mode. The sample underwent evaporation in the air like the last sample, resulting in dehydration that lasted for 14 minutes. The Stokes polarimetric images were acquired every minute to monitor the change of polarization property of the sample. The results obtained at 0 min, 7 min and 14 min are demonstrated in Fig. 6(a)-6(c)

. The change induced by dehydration can be observed in the Stokes polarimetric images, particularly the area indicated the red arrows in *S*_1_ images. The *DOPs* images of the sample representing tissue diattenuation dropped as with tissue dehydration (shown in Fig. 6(d), 6(e), 6(f)), which is consistent with the observation and analysis in the last section.

## 4. Summary

We have demonstrated a compact complete Stokes polarimetric imager that can work for multiple wavelength-bands with a frame-rate suitable for real-time applications. The imager was first validated with standard polarizing components, and was then employed as the PSA of a Mueller imaging polarimeter to image the process of dehydration and associated optical clearing of bovine tendon tissue. The results showed that the changes of polarization properties, particularly diattenuation, resulting from dehydration-induced optical clearing were clearly observed and analyzed. The imager was then used as a standalone Stokes imager to assess tissue diattenutation changes during dehydration-induced optical clearing process, and consistent trend has been observed. The results here suggested that the polarization properties of the samples rich of collagen fibres can change with the degree of dehydration. Therefore, the dehydration of the samples prepared for polarimetric imaging (e.g. polarimetric microscopy) should be carefully controlled to guarantee consistency in inter- and intra- sample inspection. It is expected that this imager could enable *in vivo* polarimetric imaging of biological tissues, and pave the way for the translation of polarimetric imaging to surgery and tissue diagnosis.

## Disclosure

The authors declare that there are no conflicts of interest related to this article.
